# Role of Renin-Angiotensin System Components in Atherosclerosis: Focus on Ang-II, ACE2, and Ang-1–7

**DOI:** 10.3389/fphys.2020.01067

**Published:** 2020-09-03

**Authors:** Gabriela M. Silva, Maria S. França-Falcão, Natália Tabosa M. Calzerra, Mickael S. Luz, Danilo Duarte A. Gadelha, Camille M. Balarini, Thyago M. Queiroz

**Affiliations:** ^1^ Laboratory of Nutrition, Physical Activity and Phenotypic Plasticity, Federal University of Pernambuco, Vitória de Santo Antão, Brazil; ^2^ Center of Biotechnology, Federal University of Paraiba, João Pessoa, Brazil; ^3^ Paraiba Higher Education Institute, IESP, Cabedelo, Brazil; ^4^ Health Sciences Center, Federal University of Paraiba, João Pessoa, Brazil

**Keywords:** angiotensin converting enzyme type 2, angiotensin II, angiotensin 1–7, atherosclerosis, endothelial dysfunction, inflammation

## Abstract

Atherosclerosis is the leading cause of vascular disease worldwide and contributes significantly to deaths from cardiovascular complications. There is a remarkably close relationship between atherosclerotic plaque formation and the activation of renin-angiotensin system (RAS). However, depending on which RAS pathway is activated, pro‐ or anti-atherogenic outcomes may be observed. This brief review focuses on the role of three of the most important pieces of RAS axis, angiotensin II (Ang-II), angiotensin converting enzyme type 2 (ACE2), and angiotensin 1–7 (Ang-1–7) and their involvement in atherosclerosis. We focused on the effects of these molecules on vascular function and inflammation, which are important determinants of atherogenesis. Furthermore, we highlighted potential pharmacological approaches to treat this disorder.

## Introduction

Cardiovascular diseases remain the leading cause of adult death worldwide (Herrington et al., 2016). Nowadays, it is already established that hypertension is a modifiable risk factor for cardiovascular diseases and the reduction in blood pressure is accompanied by a reduction in cardiovascular risk (Herrington et al., 2016). On the other hand, the persistent burden of cardiovascular events despite a highly effective control of conventional risk factors, suggests that other mechanisms might underlie a proportion of these events (Libby et al., 2019).

Atherosclerosis can be considered the primary origin of most cardiovascular diseases (Husain et al., 2015). As previously reviewed by us and by others, atherosclerosis consists of an inflammatory response of arterial wall to injuries. This inflammation is often initiated by endothelial dysfunction and progresses to cellular adhesion molecules (CAM) expression, adhesion of circulating leukocytes to the endothelial cells (Koleva et al., 2016), leucocyte migration and the formation of a fibrous cap around a lipidic core, which compromises vascular lumen (Freitas-Lima et al., 2015). In addition to its traditional role in hypertension, the long-term blood pressure control system (the renin-angiotensin system – RAS) is directly involved in the development of atherosclerotic lesions due to its mainly effects on endothelial function, inflammation, fibrosis, coagulation balance, plaque stability, and structural remodeling (Montezano et al., 2014; Husain et al., 2015).

**Figure 1:**
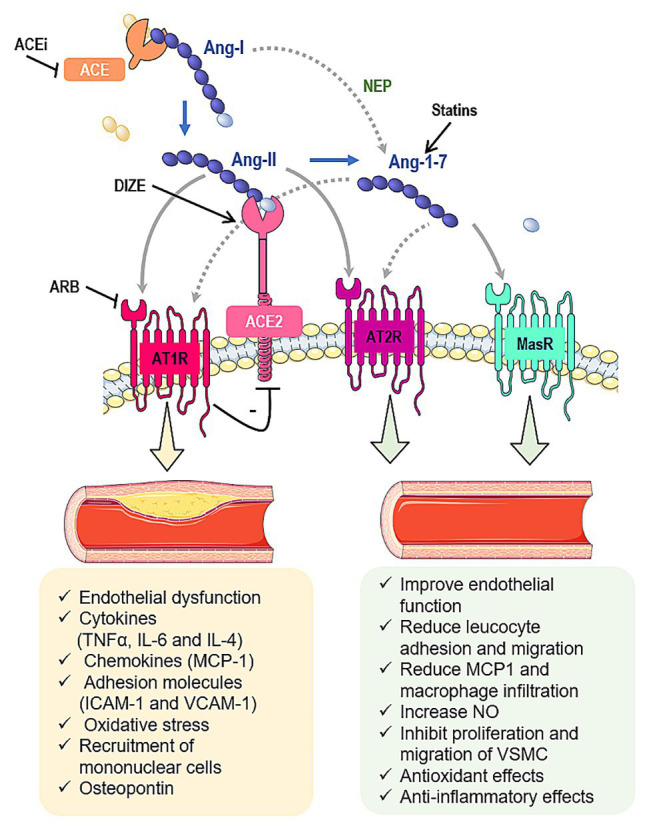
Interactions between the renin-angiotensin system (RAS) and atherosclerosis. Angiotensin II (Ang-II) is formed from the angiotensin I (Ang-I) cleavage by angiotensin converting enzyme (ACE). Ang-II can bind to Ang-II type 1 (AT1R) or type 2 (AT2R) receptors. Ang-II undergoes the action of angiotensin converting enzyme type 2 (ACE2) to be converted into angiotensin 1–7 (Ang-1-7), which classically interacts with Mas receptor (MasR). Furthermore, Ang-1–7 can bind to AT2R or it can induce the β-arrestin pathway through its interaction with AT1R. Ang-1–7 also can be produced by neprylisin (NEP) from Ang-I. In summary, the ACE/Ang-II/AT1R pathway induces atherosclerosis while the ACE2/Ang-1–7/MasR and Ang-II and Ang-1–7/AT2R pathways inhibit the atherosclerotic events. In addition, some pharmacological tools have been shown to interfere in some components of RAS cascade and prevent the atherosclerosis, such as statins, ACE inhibitors (ACEi), angiotensin receptor blockers (ARB) and diminazene aceturate (DIZE). Gray arrows indicate classic pathways while gray dotted arrows indicate alternative pathways. Black lines indicate potential pharmacological approaches to stimulate/increase (arrows) or block/decrease (lines) components of RAS.

Along with the classic cascade in RAS, which involves the conversion of angiotensinogen to angiotensin I (Ang-I) by renin, followed by its cleavage to angiotensin II (Ang-II) by angiotensin converting enzyme (ACE), other peptides and enzymes related to RAS are important in atherogenesis (Figure 1; Montezano et al., 2014). In this context, we highlight the role of the angiotensin converting enzyme type 2 (ACE2), which is typically responsible to form angiotensin 1–7 (Ang-1–7) from Ang-II. The heptapeptide is described to oppose Ang-II effects by mediating vasodilation, growth-inhibition, anti-inflammatory responses, and anti-thrombotic effects (Montezano et al., 2014). Considering that, this review is devoted to summarize the effects of Ang-II, ACE2, and Ang-1–7 in atherosclerosis, highlighting the promising interventions that could lead to RAS modulation and atherosclerosis treatment.

## Angiotensin II and Atherosclerosis

Ang-II is the main effector of RAS (Colafella et al., 2019). The effects of Ang-II are mediated by its binding into the angiotensin type 1 and type 2 receptors (AT1R and AT2R, respectively). These receptors are G protein-coupled receptors that tend to present opposing activities (Kellici et al., 2015). AT1R is primarily responsible for the classic pro-hypertensive activity of Ang-II, whereas the AT2R is reported to present antagonistic effects to the AT1R (Figure 1; Ding et al., 2016).

It has been shown that Ang-II directly induces endothelial dysfunction and increases endothelial oxidative stress through the production of reactive oxygen species (ROS) such as superoxide anions (O_2_^−^) derived from the complex enzyme nicotinamide adenine dinucleotide phosphate oxidase (NADPH oxidase). This occurs predominantly through interaction with endothelial AT1R (Ziegler et al., 2020), which mediates increase in Ca^2+^ concentration in endothelial cells, promoting activation of calmodulin and interaction with the Nox5/Ca^2+^ calmodulin binding domain (Montezano et al., 2010; Piqueras and Sanz, 2020). Nox5 is a member of the NADPH oxidase family which is not found in rodents but is highly expressed in coronary arteries obtained from individuals with coronary artery disease (Guzik et al., 2008; Gray and Jandeleit-Dahm, 2015). In atherosclerosis, oxidative and inflammatory processes involve increased expression and activation of Nox5 in both vascular cells and resident macrophages (Touyz et al., 2019).

**Figure 2:**
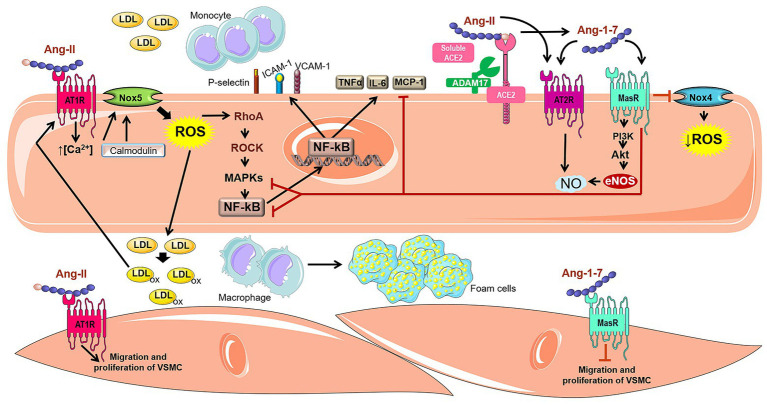
Involvement of Ang-II, ACE2, and Ang-1–7 in atherogenic pathways. The Ang-II binding into AT1R can activate Nox5 through a calcium/calmodulin-dependent pathway. The activated Nox5 induces the formation of ROS and stimulates the RhoA/ROCK pathway, which in turn, activates MAPKs and induces the transactivation of several transcription factors such as NF-κB. The expression of several genes is regulated by NF-κB, for instance cytokines (TNF-α and IL-6), chemokines (MCP-1), adhesion molecules (P-selectin, ICAM-1 and VCAM-1), which are involved in Ang-II-induced migration of mononuclear leukocytes. In addition, Ang-II is cleaved by ACE2 and produces Ang-1–7, an important RAS counter-regulator. Ang-1–7 shows the potential to negatively regulate atherogenic pathways, inducing anti-inflammatory effect, weakening monocyte migration and decrease of vascular lipids accumulation. These actions attributed to Ang-1–7 are related to the reduction of oxidative stress and the synthesis of inflammatory cytokines due to inhibition of the Nox4 and NF-κB-mediated pathways. Furthermore, Ang-1–7 stimulates the PI3K/Akt pathway, leading to phosphorylation of eNOS and NO formation, which improves the endothelial function. Ang-1–7 is also capable of promoting endothelial activation of AT2R, which also stimulates the NO cascade. In VSMC, Ang-1–7 inhibits muscle cell migration and proliferation, in contrast to Ang-II which possess proliferative and hypertrophic effects.

Activation of Nox5 mediated by Ang-II produces O_2_^−^, activates RhoA and leads to the subsequent stimulation of Rho-associate kinase in human umbilical arterial endothelial cells culture (Escudero et al., 2015). The RhoA/ROCK pathway is an upstream regulator of mitogen-activated protein kinases (MAPKs – p38MAPK and ERK1/2), which promotes transactivation of several transcription factors, including NF-κB (Piqueras and Sanz, 2020). NF-κB regulates the expression of numerous genes, such as cytokines, tumor necrosis factor alpha (TNF-α) and interleukin 6 (IL-6), chemokines (monocyte chemoattractant protein – MCP-1), adhesion molecules (P-selectin, ICAM-1, and VCAM-1), the inflammatory enzyme cyclooxygenase type 2 (COX-2), and angiotensinogen (Durante et al., 2012; Liang et al., 2015). Moreover, activation of NF-κB seems to be an important signal transducer involved in the upregulation of oxidized low-density lipoprotein (ox-LDL)–mediated AT1R expression (Figure 2; Li et al., 2000).

It is likely that TNF-α, released upon Ang-II stimulation of the AT1R, in combination with IL-4 acts as a paracrine molecule, inducing selective adhesion of mononuclear cells to the arterial endothelium through increased expression of CAM, and the release of varied chemokines involved in the recruitment of mononuclear cells (Piqueras and Sanz, 2020). Shu et al. (2019) demonstrated that Ang-II induces monocyte chemotactic protein-induced protein expression (MCPIP1) through an AMPK/p38 MAPK dependent pathway. The increase in MCPIP1 expression triggered apoptosis in macrophages, contributing to atherosclerotic plaque vulnerability.

In addition, Ang-II induces the expression of osteopontin, a multifunctional protein found in many cell types, including macrophages, endothelial cells, smooth muscle cells (SMCs), and epithelial cells. Osteopontin is found in atherosclerotic lesions, especially in association with macrophages and foam cells, suggesting that this protein plays an important role in the development and progression of atherosclerosis (Ding et al., 2016). The molecular mechanisms related to osteopontin involve recruitment of inflammatory cells and migration of foam cells through the binding to integrins (Giachelli and Steitz, 2000).

Ang-II also up-regulates the LOX-1 gene. LOX is a transmembrane glycoprotein that serves as a receptor for oxidized LDL (Lubrano and Balzan, 2016). In the endothelium, binding of oxLDL to LOX-1 causes increase in leukocyte adhesion molecules, activates apoptosis pathways, increases ROS and induces endothelial dysfunction. In a pro-inflammatory environment, LOX-1 is positively regulated in macrophages and is associated with more than 40% of oxLDL uptake, contributing to the formation of foam cells (Kattoor et al., 2019). In addition, oxLDL increases the generation of ACE, which in turn induces the Ang-II formation. This octapeptide increases the expression of LOX-1, which positively regulates the expression of AT1R, contributing to a self-perpetuating pro-atherogenic cycle. It has also been reported that ACE inhibitors and AT1R blockers (ARBs) decrease the expression of LOX-1 (Lubrano and Balzan, 2016).

According to experimental and clinical data, ACE inhibitors and ARBs appear to have beneficial anti-atherosclerotic effects (Tousoulis et al., 2015). Studies have shown that enalapril ameliorated oxidative vascular injury, suppressed NADPH oxidase activity, decreased inflammatory mediators and regulated the antioxidant defense system in apolipoprotein E-deficient mice (ApoE-KO; Suarez-Martinez et al., 2014; Husain et al., 2015), an animal model commonly used to study atherosclerosis.

It has been shown that the ARB olmesartan significantly reduced vascular inflammation in hypertensive patients, with a significant reduction in serum levels of many inflammatory markers, such as C-reactive protein, TNF-α, IL-6, and MCP-1 (Fliser et al., 2004; Durante et al., 2012). Moreover, long-term therapy with valsartan has been associated with atherosclerosis regression in individuals with thickening of the carotid wall. These effects were accompanied by concomitant improvements in oxidative stress markers, inflammation, and peripheral smooth muscle function (Ramadan et al., 2016).

## ACE2 and Atherosclerosis

The first evidence of a relationship between ACE2 and atherosclerosis was demonstrated by Zulli et al. (2006). They have shown the immunolocalization of ACE2 protein in macrophages and SMC actin-positive cells from rabbit atherosclerotic plaques. After this study, several experimental and clinical evidence have confirmed the involvement of ACE2 in atherosclerosis, suggesting its anti-atherogenic role (Dong et al., 2008; Lovren et al., 2008).

Dong et al. (2008) have found that ACE2 overexpression on aortic plaques attenuate the progression of early lesions in rabbits that underwent to endothelial injury and received atherogenic diet, probably by conversion of Ang-II to Ang-1–7. In this scenario, there was a reduction in local inflammation, lipid deposition, macrophage infiltration, and MCP-1 expression, in addition to an increase in collagen content, resulting in stabilized plaques. Similar results were found in rabbits fed with a high-cholesterol diet. The anti-atherosclerotic effects of ACE2 were associated with inhibition of proliferation and migration of vascular SMC and improvement of endothelial function. Additionally, ACE2 produced down-regulation of ERK1/2, p38 MAPK, JAK-STAT, and Ang-ll/ROS/NF-κB signaling pathways and upregulation of the PI3K-Akt pathway (Zhang et al., 2010).

Likewise, overexpression of ACE2 in ApoE-KO mice attenuated atherosclerotic lesion size and improved endothelial homeostasis, at least in part, through a mechanism that involves reduction of Ang-II-induced ROS generation (Lovren et al., 2008). In accordance to these data, Zhang et al. (2015a) also have shown that inhibition of inflammatory response, such as reduction of Ang-II-induced expression of adhesion molecules and cytokines prevent atherosclerotic plaque evolution in ApoE-KO animals overexpressing ACE2.

The protective role of ACE2 on atherosclerosis was also supported by the use of ACE2-deficient mice model (ACE2-KO). ACE2-deficiency in both LDL receptor-deficient mice (LDLR-KO) and ApoE-KO backgrounds resulted in larger atherosclerotic lesions when compared to their respective controls. Furthermore, the increased atherosclerotic vulnerability was associated to intraplaque inflammatory profile (Thomas et al., 2010; Thatcher et al., 2011; Sahara et al., 2014). On the other hand, the protective role of ACE2 on atherosclerosis in humans is not well-established yet.

In 2008, Sluimer and colleagues demonstrated the presence of ACE2 in humans. They detected ACE2 protein in human veins, healthy and atherosclerotic arteries, expressed in endothelial cells, SMCs, and macrophages. In addition, they found ACE2 messenger RNA (mRNA) and protein in early and advanced atherosclerotic lesion from humans. Despite total protein expression of ACE2 was similar during all stages of atherosclerosis, ACE2 activity was lower in advanced lesions, suggesting differential regulation of ACE2 in progression of atherosclerosis (Sluimer et al., 2008).

Anguiano et al. (2016) have found that baseline circulating ACE2 activity was enhanced in chronic kidney disease patients with atherosclerotic plaques when compared to patients with no plaque, suggesting that higher circulating ACE2 activity is associated with higher risk for silent atherosclerosis. Accordingly, Zhou et al. (2020) have shown an increase in circulating ACE2 protein levels in women with coronary heart disease (CHD) when compared to healthy group. This increase was associated with multi-vessel lesions, corroborating with the reports by Anguiano et al. (2016) and indicating the ACE2 as a compensatory mechanism in coronary atherosclerosis.

ACE2 is an integral cell membrane protein that can undergo cleavage or shedding and release its catalytically active ectodomain into surrounding milieu. The main promoter of ACE2 shedding is A Disintegrin and Metalloprotease 17 (ADAM17), which has been involved in atherosclerosis (Canault et al., 2006, 2007). This evidence and the results found by Zhou et al. (2020) allowed these authors to conclude that the increase in circulating ACE2 level is due to increasing tissue ACE2 synthesis from mRNA and augmented ACE2 protein shedding followed by its increase in circulation. All together these data show the increased circulating ACE2 protein levels or activity as biomarkers of atherosclerosis and encourage further studies in this direction.

Some therapeutic strategies for atherosclerosis targeting ACE2 have been thought, either with new drugs or drugs already used in the clinic. A recent study has demonstrated that overexpression of ACE by plasmid-mediated transfection in both primary monocytes and THP-1 cells leads to a marked decrease of ACE2 mRNA expression and induces a pro-atherogenic phenotype with elevated gene expression of the cellular adhesion molecules ICAM-1, VCAM-1, and macrophage colony-stimulating factor (MCSF). All these effects were partly reversed by captopril and losartan (Trojanowicz et al., 2017).

In that context, Zhang et al. (2015b) have shown that losartan inhibited the evolution of atherosclerotic plaques in high-cholesterol fed rabbits as well as increased the ACE2 protein expression in the plaques. In addition, Ang-II downregulated ACE2 protein expression and activity in SMC cell culture and losartan significantly blocked Ang-II-induced reduction of both ACE2 protein and activity. These data indicate that Ang-II generation by ACE can affect the expression and activity of ACE2 and ACE inhibitors or AT1R antagonists can upregulate ACE2 and favor its anti-atherogenic effects.

ACE2-activating drugs also seem promising, with emphasis on diminazene aceturate (DIZE) (Qaradakhi et al., 2020), which has several protective effects, such as improvement of metabolic profile and reduction of lipogenesis in mice (Macedo et al., 2015), anti-hypertensive effects in renovascular hypertensive rats (De Maria et al., 2016), and improvement of pulmonary endothelial function in Sprague Dawley rats (Shenoy et al., 2013). Thatcher et al. (2014) have found that DIZE decreases formation and severity of Ang-II-induced abdominal aortic aneurysms (AAA). Ang-II-induced AAA is characterized by progressive leukocyte accumulation, extracellular matrix degradation, luminal expansion, and thrombus (Saraff et al., 2003), being closely related to atherosclerosis. In addition, Fraga-Silva et al. (2015) have demonstrated that DIZE enhances the stability of atherosclerotic plaques in ApoE-KO mice and reduces the expression of ICAM-1 and VCAM-1. Although the mentioned studies have been performed on animal models, they suggest DIZE as a potential drug for the treatment of atherosclerosis and related cardiovascular diseases.

## Angiotensin 1–7 and Atherosclerosis

Ang-1–7 was investigated three decades ago as an important counter-regulator component of RAS, promoting hypotension and bradycardia after microinjection in dorsal motor nucleus of the vagus (Santos et al., 1988; Campagnole-Santos et al., 1989). The classical formation of Ang-1–7 occurs through ACE2 action on Ang-II. Alternatively, Ang-1–7 is formed by the cleavage of Ang-1–9 facilitated by ACE. Moreover, Ang-I can be directly converted into Ang-1–7 by action of neutral endopeptidase (neprylisin – NEP; Santos et al., 2003; Santos, 2014).

The formation of Ang-1–7 in vascular endothelium was first identified by Santos et al. (1992) using human aortic and human umbilical vein endothelial cells (HUVEC; Santos et al., 1992). Robust studies have shown that Ang-1–7 induces MasR activation, a G protein-coupled receptor which stimulates the PI3K/Akt pathway leading to phosphorylation of endothelial nitric oxide (NO) synthase and consequent NO production and releasing (Sampaio et al., 2007). Of note, Ang-1–7 is able to promote AT2R endothelial activation, which stimulates the bradykinin–NO cascade (Walters et al., 2005; Villela et al., 2015). NO is one of the most important factors released by endothelium. This gas is involved in vascular homeostasis and its decrease induces endothelial dysfunction (Cheng et al., 2009; Forstermann and Sessa, 2012), which is the key factor in atherogenesis (Qaradakhi et al., 2016).

Studies have demonstrated that Ang-1–7 stimulates endothelial cells function restoration by increasing NO bioavailability (Pignone et al., 2007; Sampaio et al., 2007). In addition, Ang-1–7 downregulates adhesion molecules such as VCAM-1 and ICAM-1 in endothelium by preventing both the phosphorylation of p38 MAPK and the expression of NF-κB (Anton et al., 2007; Zhang et al., 2013; Liang et al., 2015). Moreover, Ang-1–7 induces proliferation of endothelial progenitor cells in the injured vascular tissue triggered by atherogenesis (Wang et al., 2010; Zhang et al., 2015c).

During the vascular inflammation, many cytokines and inflammatory cells are required to begin and maintain atherosclerosis progression. In this context, Ang-1–7 has been described to induce anti-inflammatory phenotypes which contribute to restrain vascular lipid accumulation (Yang et al., 2013; Jiang et al., 2014). Yang et al. (2015a) found that Ang-1–7 treatment reduced the oxidative stress and macrophage infiltration due to decreasing in Nox4 (a subunit of NADPH oxidase complex) and NF-κB in aorta from ApoE-KO (Figure 2; Yang et al., 2015a). Another interesting study revealed that Ang-1–7 administration induced a remarkable decrease in the expression of pro-inflammatory cytokines such as IL-6, TNF-α, and MCP-1 in both aortic plaque and macrophages from ApoE-KO (Yang et al., 2013). Furthermore, in the same mouse model, pretreatment with AVE0991, a MasR agonist, reduced activated CD4^+^ T cells (Jawien et al., 2012a) and IL-12 (Jawien et al., 2012b). All these findings corroborate with an anti-inflammatory effect of Ang-1–7/MasR pathway in atherosclerosis.

In contrast to Ang-II-induced proliferative and hypertrophic effects, Ang-1–7 inhibits the migration and proliferation of vascular SMCs (Jiang et al., 2014; McKinney et al., 2014). This effect was described by Yang et al. (2013), showing that Ang-1–7 induces activation of MasR/ERK1,2/p38 and MasR/JAK/STAT pathways in vascular SMCs to mitigate the atherosclerotic plaque formation (Yang et al., 2013). Furthermore, Ang-1–7 has demonstrated a potential to negatively regulate the vascular fibrosis, as can be noticed by decreasing in matrix metalloproteases (MMP) MMP-2/MMP-9 in atherosclerotic plaques (Yang et al., 2013). Accordingly, Ang-1–7 treatment promoted a reduction in the neointimal layer growth by structural recovery of endothelium and showed atheroprotective properties attributed to its binding to both AT2R and MasR (Faria-Silva et al., 2005; Tesanovic et al., 2010). In addition, Ang-1–7 reduced atherosclerotic lesion formation by decrease in collagen accumulation through activation of AT2R (Dandapat et al., 2008). Conversely, it was described an increase in collagen content after Ang-1–7 administration, resulting in the increase of plaque stability (Yang et al., 2013). Similarly, the treatment with an Ang-1–7 antagonist, A779, induced a decline in plaque stability and reduction in collagen level (Yang et al., 2015b). Moreover, the heptapeptide can play a role as a β-arrestin-biased AT1R agonist without induce the Gq subunit activation, suggesting an additional antihypertrophic effect attributed to this peptide (Teixeira et al., 2017; Paz Ocaranza et al., 2020)

Interestingly, increase in plasmatic Ang-1–7 has been involved in regulation of lipid metabolism. It promoted a reduction in triglycerides and cholesterol levels, together with a decrease in adipose tissue mass as well as an improvement of glucose metabolism (Santos et al., 2010). The authors have suggested an involvement of adiponectin in the regulation of the glucose and lipid metabolism induced by Ang-1–7 (Santos et al., 2010). Curiously, the knocking out of MasR promoted opposing effects, once it augmented cholesterol and triglycerides levels and worsened the carbohydrate metabolism (Santos et al., 2008).

## The Role of Statins on RAS Components and Atherosclerosis

Some therapeutic strategies have been validated to positively modulate the RAS. The statins, 3-hydroxy-3-methyl-glutaryl-coenzyme A reductase (HMGcoA-reductase) inhibitors, have emerged due to its pleiotropic properties demonstrating additional effects apart from those of decreasing cholesterol levels (Zhang et al., 2015c). Treatment with statins such as atorvastatin and rosuvastatin have promoted an upregulation of ACE2/Ang-1–7 axis, reducing the proliferation of vascular SMCs and intimal thickening, respectively (Li et al., 2000; Suski et al., 2014), effects that are closely related to atherogenesis. The mechanisms by which the statins act to promote these effects are still unclear; however, studies have revealed that HMGcoA-reductase inhibitors decrease the activation of NF-κB induced by TNF-α and Ang-II, factors responsible to stimulate the migration and proliferation of vascular wall (Ortego et al., 1999; Friedrich et al., 2006; Tristano et al., 2007; Suski et al., 2014). Furthermore, authors have demonstrated that atorvastatin induced an increase in ACE2 protein expression in heart and kidney from high cholesterol-fed rabbits and augmented the occupancy of histone H3 acetylation (H3-Ac) mark on ACE2 promoter region in heart, demonstrating direct or indirect ACE2 epigenetic upregulation (Tikoo et al., 2015).

The role of statins on RAS components also have been observed in clinical trials as showed by Schindler et al. (2014) that identified, for the first time, an increase of Ang-1–7 level in hypercholesterolemic subjects after atorvastatin treatment. Altogether, those responses suggest an important role of statins on RAS components, including a decrease in Ang-II and, apparently, an upregulation in the ACE2/Ang-1–7 axis. This fact could be crucial to the atherosclerosis and cardiovascular diseases therapy.

## Conclusion

In conclusion, here we briefly reviewed the role played by RAS components such as Ang-II, ACE2, and Ang-1–7 in atherosclerosis development. According to what is expected to components of RAS, Ang-II is considered to have pro-atherogenic effects while ACE2 and Ang-1–7 anti-atherogenic profiles. In addition to the direct pressure-related roles of these peptides, their effects on atherosclerosis involve modulation of endothelial function, oxidative stress, inflammation, cellular migration and proliferation, as well as plaque stability. Pharmacological strategies currently used to modulate the pressor effects of RAS components can offer beneficial outcomes in atherosclerosis. Moreover, we highlight the role played by statins, which have been identified to increase the RAS compensatory components (ACE2 and Ang-1–7), and induce an additional effect against the plaque formation. For this reason, the HMGcoA-reductase inhibitors should be considered when clinical decisions are made.

## Author Contributions

CB, TQ and MF-F conceived the manuscript and revised it critically. CB and NC prepared the figures. GS, NC, ML and DG drafted the manuscript. All authors contributed to the article and approved the submitted version.

### Conflict of Interest

The authors declare that the research was conducted in the absence of any commercial or financial relationships that could be construed as a potential conflict of interest.
